# A 1232 bp upstream sequence of glutamine synthetase 1b from *Eichhornia crassipes* is a root-preferential promoter sequence

**DOI:** 10.1186/s12870-021-02832-x

**Published:** 2021-01-29

**Authors:** Yanshan Zhong, Xiaodan Lu, Zhiwei Deng, Ziqing Lu, Minghui Fu

**Affiliations:** Bioengineering Department, Biological and Pharmaceutical College, Guangdong University of Technology, Guangzhou, Guangdong P.R. China 510006

## Abstract

**Background:**

Glutamine synthetase (GS) acts as a key enzyme in plant nitrogen (N) metabolism. It is important to understand the regulation of GS expression in plant. Promoters can initiate the transcription of its downstream gene. *Eichhornia crassipes* is a most prominent aquatic invasive plant, which has negative effects on environment and economic development. It also can be used in the bioremediation of pollutants present in water and the production of feeding and energy fuel. So identification and characterization of GS promoter in *E. crassipes* can help to elucidate its regulation mechanism of GS expression and further to control its N metabolism.

**Results:**

A 1232 bp genomic fragment upstream of *EcGS1b* sequence from *E. crassipes* (*EcGS1b*-P) has been cloned, analyzed and functionally characterized. TSSP-TCM software and PlantCARE analysis showed a TATA-box core element, a CAAT-box, root specific expression element, light regulation elements including chs-CMA1a, Box I, and Sp1 and other cis-acting elements in the sequence. Three 5′-deletion fragments of *EcGS1b* upstream sequence with 400 bp, 600 bp and 900 bp length and the 1232 bp fragment were used to drive the expression of β-glucuronidase (GUS) in tobacco. The quantitative test revealed that GUS activity decreased with the decreasing of the promoter length, which indicated that there were no negative regulated elements in the *EcGS1*-P. The GUS expressions of *EcGS1b*-P in roots were significantly higher than those in leaves and stems, indicating *EcGS1b*-P to be a root-preferential promoter. Real-time Quantitative Reverse Transcription-Polymerase Chain Reaction (qRT-PCR) analysis of *EcGS1b* gene also showed higher expression in the roots of *E.crassipes* than in stems and leaves.

**Conclusions:**

*EcGS1b*-P is a root-preferential promoter sequence. It can specifically drive the transcription of its downstream gene in root. This study will help to elucidate the regulatory mechanisms of *EcGS1b* tissue-specific expression and further study its other regulatory mechanisms in order to utilize *E.crassipes* in remediation of eutrophic water and control its overgrowth from the point of nutrient metabolism.

## Background

*Eichhornia crassipes* is a most prominent aquatic invasive plant [1], with negative effects on environment and economic development [2, 3]. Also, it is regarded as a valuable resource with several unique properties, and previous studies have reported that *E. crassipes* had high absorption efficiency of nitrogen (N), phosphorus (P) and heavy metals [4–6]. So it was used in the bioremediation of pollutants present in water [7, 8]. Furthermore, *E. crassipes* served as a good economic raw material for the production of feeding and energy fuel [9–11]. There was about 33% of crude protein accumulation in the leaves when grown in sewage wastewater, and it was also used in the production of fuels such as ethanol and methane along with some microorganisms [12, 13]. Thus, studying the biochemical metabolism of *E. crassipes* from the molecular level assists in further utilizing and controlling this weed.

Glutamine synthetase (GS) in plants acts as a key enzyme in N metabolism, converting inorganic N (NH_4_^+^ or NO_3_^−^) that is absorbed from outside into organic N and further incorporating into other biomacromolecules by GS (Glutamine synthetase)-GOGAT (Glutamate Synthase) cycle [14]. GS could be divided into GS1, GS2 and GS3 according to its protein structure and gene sequence [15, 16], and higher plants have GS1 and GS2 forms in roots and leaves. GS2 is generally present in the leaf tissues, while GS1 in the roots and vascular tissues [11, 17]. Many studies have shown that GS1 in roots assimilates NH_4_^+^ from soil into the plant and GS1 in leaves reassimilates NH_4_^+^ generated during protein turnover in leaves, while GS2 assimilates NH_4_^+^ derived from photorespiration and nitrate reduction [18]. GS plays an important role in N assimilation which is essential to the plant growth and development. Plants have a refined regulation mechanism of GS expression, which is closely coordinated with the external condition and the development status of plants. Zhang et al. reported that the regulation of GS isozymes might promote flow strength and enhance N use efficiency (NUE) by a complex C-N metabolic mechanism [19]. In wheat, GS1;1 expression was upregulated in response to a reduction in N supply [20], whereas high NH_4_^+^ supply specifically induced the expression of the GS1–3 isogene in barley and sorghum [21, 22]. GS expression was regulated by the external N application, but the extent of this regulation depended on the plant species, N source and plant tissue [18]. What is the regulation mechanism of GS expression? The study on the promoter of GS gene could reveal the temporal and spatial properties of GS expression, and further reveal the molecular mechanism of GS regulation at the transcriptional level, which could be better utilized to improve the absorption and utilization of N by plants.

Promoters are upstream sequences of the 5′ end of genes that regulates gene expression, contains RNA polymerase and transcriptional factor recognition and binding sites, and enables the initiation of transcription. Promoter sequences included core elements and regulatory elements, and the transcriptions of their downstream genes could be regulated by the conditions corresponding to these regulatory elements [23–25]. Heat shock elements of the soybean Gmhsp 17.3-B gene were involved in heat shock promoter activation during tobacco seed maturation [26]. The expression of isopentenyltransferase driven by the cold inducible AtCOR15a promoter could provide the sugarcane cultivated in tropical and subtropical region a greater tolerance to cold stress [27]. The root-specific promoter PsPR10 from *Pinus strobus* containing many abiotic regulatory elements could efficiently initiate the expression of downstream genes in root under the different hormonal or salt stress conditions [28]. So the study of the promoter and its regulation can help to elucidate the expression mechanism of its downstream gene. In this study, we isolated and characterized the promoter sequence of *EcGS1b* gene. For investigating the function of *EcGS1b*-P, transgenic tobaccos with GUS driven by *EcGS1b*-P were developed and it was found that *EcGS1b*-P could drive the gene expression preferentially in roots. To further validate this, the *EcGS1b* gene expression levels were measured by using a real-time qRT-PCR.

## Results

### Cloning and analysis of *EcGS1b*-P

**Fig. 1:**
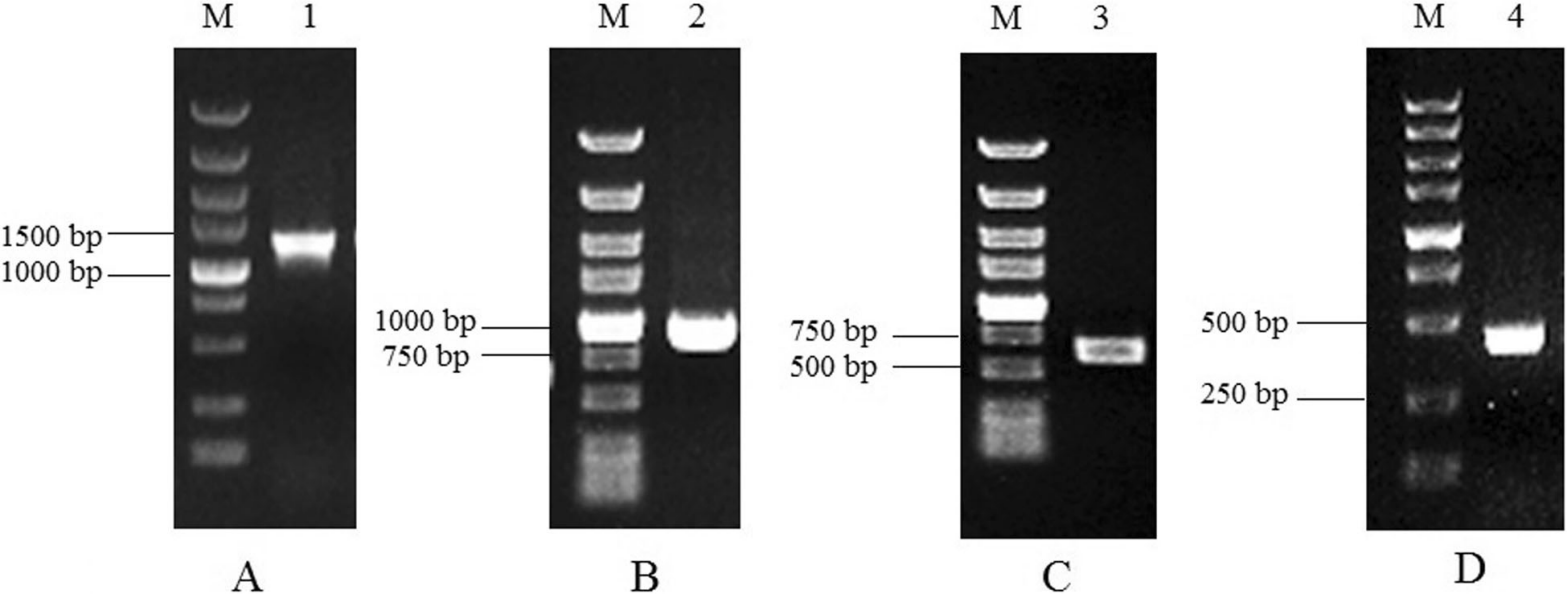
PCR amplification products of promoters. M: DL5000Marker; 1: p1200; 2: p900; 3: p600; 4: p400

**Fig. 2:**
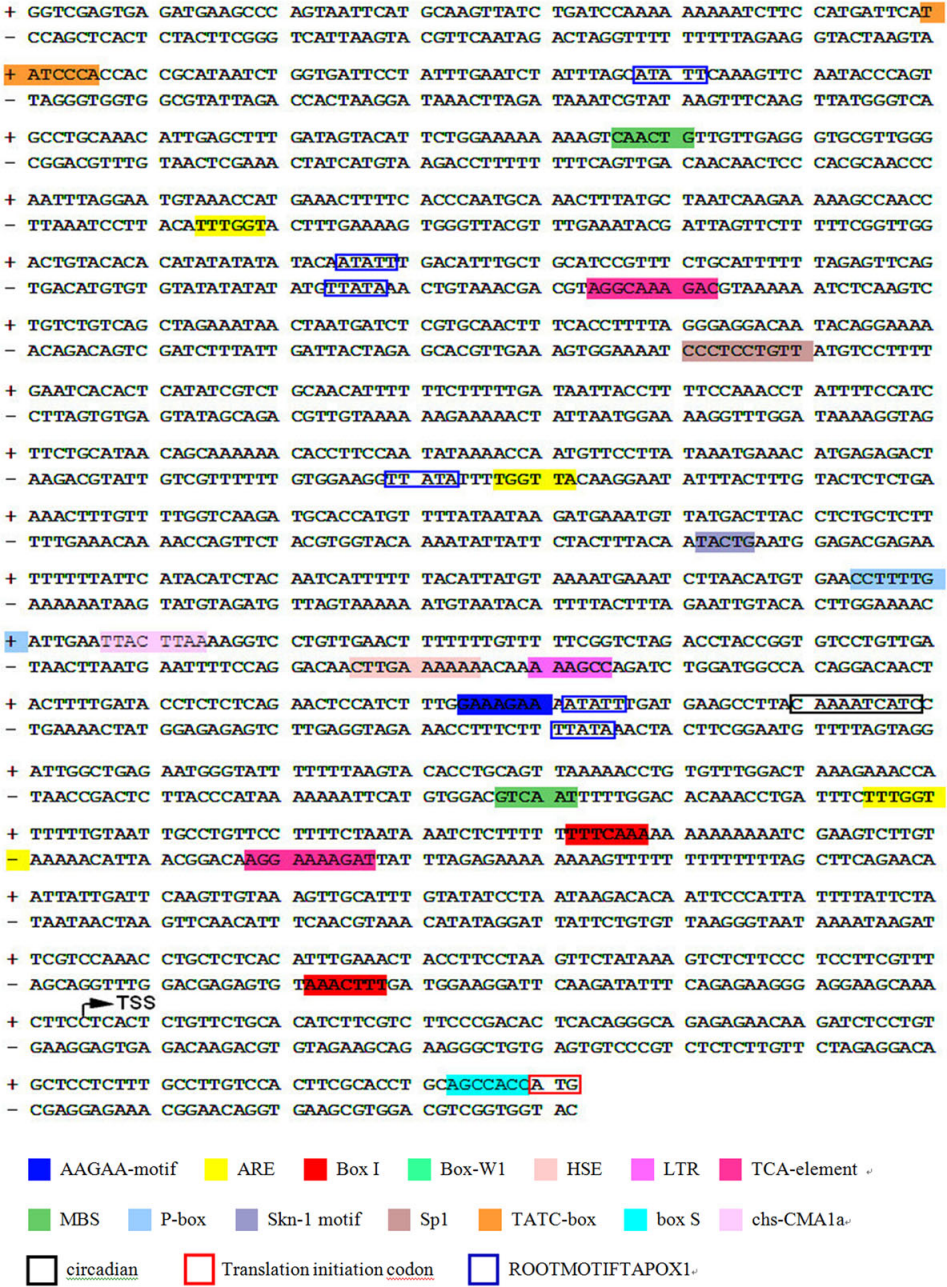
Sequence analysis of gene *EcGS1b* by TSSP-TCM software and PlantCARE

To clone the regulatory regions of *EcGS1b* gene, the primer pairs were designed from the corresponding cDNA sequences. Using PCR gene walking on genomic DNA from *E. crassipes*, the upstream of *EcGS1b* gene was cloned thrice with the nested-PCR (Fig. 1) and then was sequenced. The *EcGS1b* gene upstream sequence (1232 bp) was obtained and named as *EcGS1b*-P. This sequence was submitted to NCBI database and assigned the accession number MT154418. Homology search using Blast programs revealed no similarities of known genes or promoters in the GenBank database. The transcriptional start site (TSS) was started from the 105th bp upstream to the ATG codon. The TSS distances from the TATA-box (− 30 bp) and the CAAT-box (− 96 bp) were consistent with those that were usually described for other plant promoters [29, 30]. Table 1 was the predicted result using TSSP-TCM software and PlantCARE, showing that some cis-acting elements involved in abiotic stress tolerance (MBS, HSE, LTR, circadian), endosperm and root specific expression elements (Skn-1_motif, ROOTMOTIFTAPOX1), light regulatory elements (chs-CMA1a, Box I and Sp1), and salicylic acid and gibberellin-responsive elements (TCA-element, P-box) existed in this sequence (Fig. 2). So *EcGS1b*-P contained the core promoter, tissue specific expression elements, light regulation elements and other cis-acting elements, which might regulate the time and the space of GS1b expression.

**Table 1:** Functions of promoter elements

Site names	Position	Strand	Function
TATC-box	− 1159	+	cis-acting element involved in gibberellin reaction
MBS	− 1043	+	MYB binding site involved in drought induction
MBS	− 352	–	MYB binding site involved in drought induction
P-box	− 535	+	gibberellin response element
chs-CMA1a	−522	+	part of a light response element
Box I	− 277	+	light response element
Box I	−159	–	light response element
circadian	− 399	+	cis-acting regulatory element of circadian rhythm
TCA-element	− 905/−301	–	cis-acting element involved in salicylic acid response
Sp1	− 828	–	light response element
Skn-1_motif	− 617	–	cis-acting regulatory element of endosperm expression
HSE	− 503	–	cis-acting element involved in heat stress response
LTR	− 489	–	cis-acting element involved in low-temperature response
Box-W1	−656	–	cis-acting element involved in fungu
ROOTMOTIFTAPOX1	− 1111/− 924/− 417	+	root-specific expression element
ROOTMOTIFTAPOX1	− 925/− 710/− 418	–	root-specific expression element

Note: The A in ATG start codon was defined as + 1

### Histochemical analysis of GUS expressions driven by different length promoters of *EcGS1b*-P

**Fig. 3:**
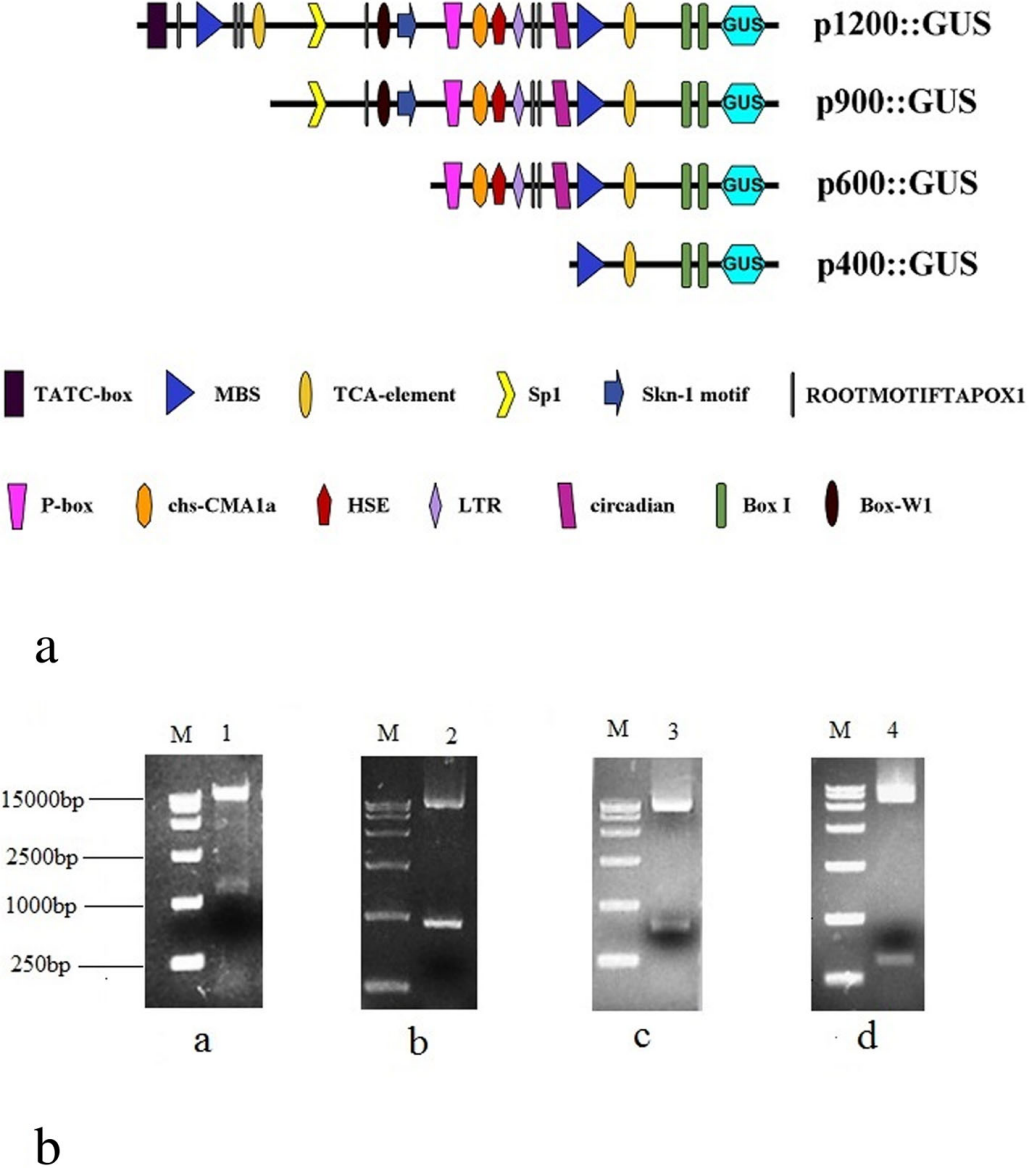
Construction of four promoters with different length. **a** Diagram of promoters with different function elements and its driven GUS. **b** Enzymes digestion test of promoter expression vector for plants. M: DL15000Maker; 1: Enzymes digestion test of p1200::GUS; 2: Enzymes digestion test of p900::GUS; 3: Enzymes digestion test of p600::GUS; 4: Enzymes digestion test of p400::GUS

**Fig. 4:**
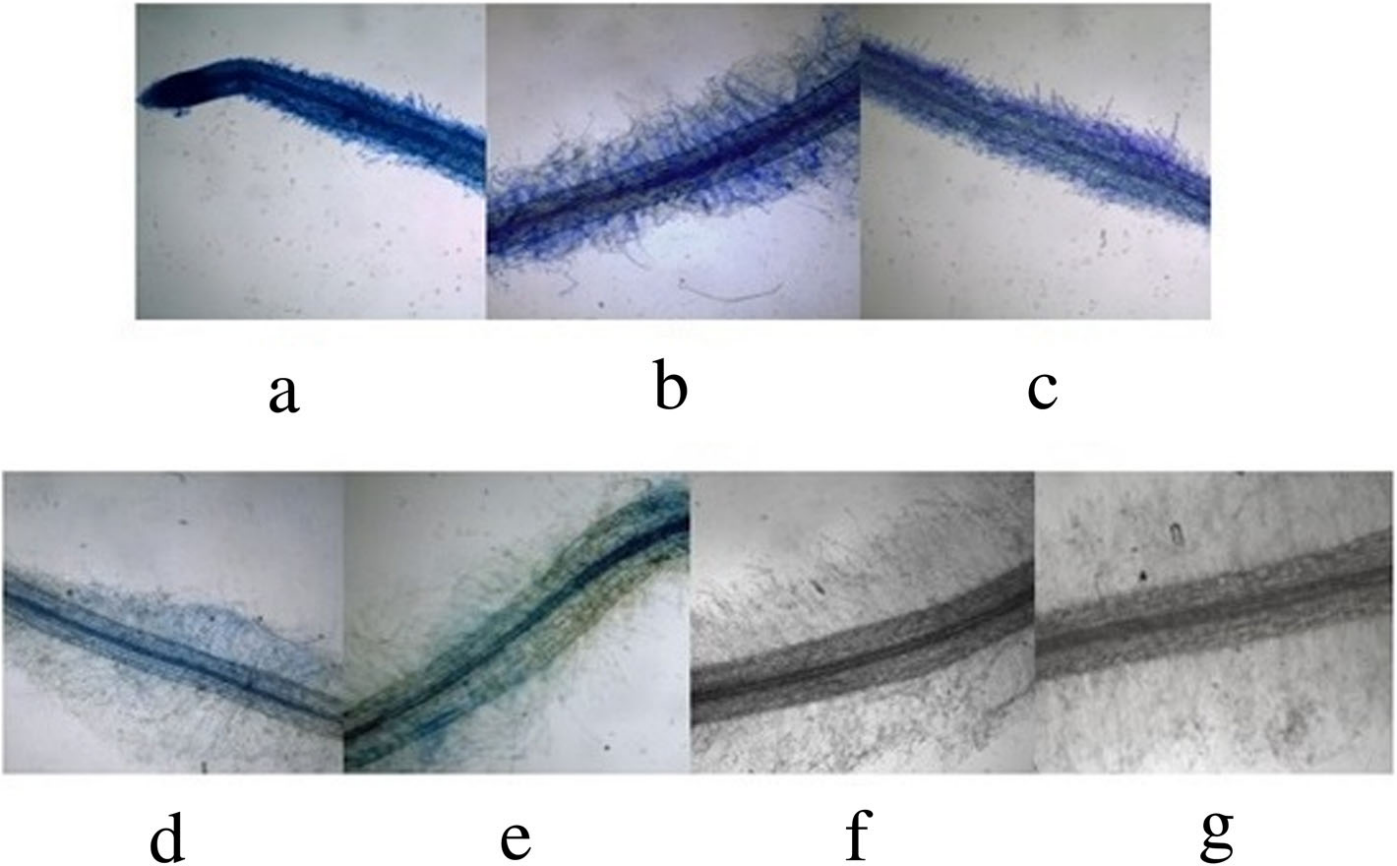
GUS expression in transgenic tobacco root. **a** The tobacco root transformed with plasmid pBI121; **b** The tobacco root transformed with plasmid p1200::GUS; **c** The tobacco root transformed with plasmid p900::GUS; **d** The tobacco root transformed with plasmid p600::GUS; **e** The tobacco root transformed with plasmid p400::GUS; **f** The tobacco root transformed with plasmid pBI101; **g** The wild tobacco root

**Fig. 5:**
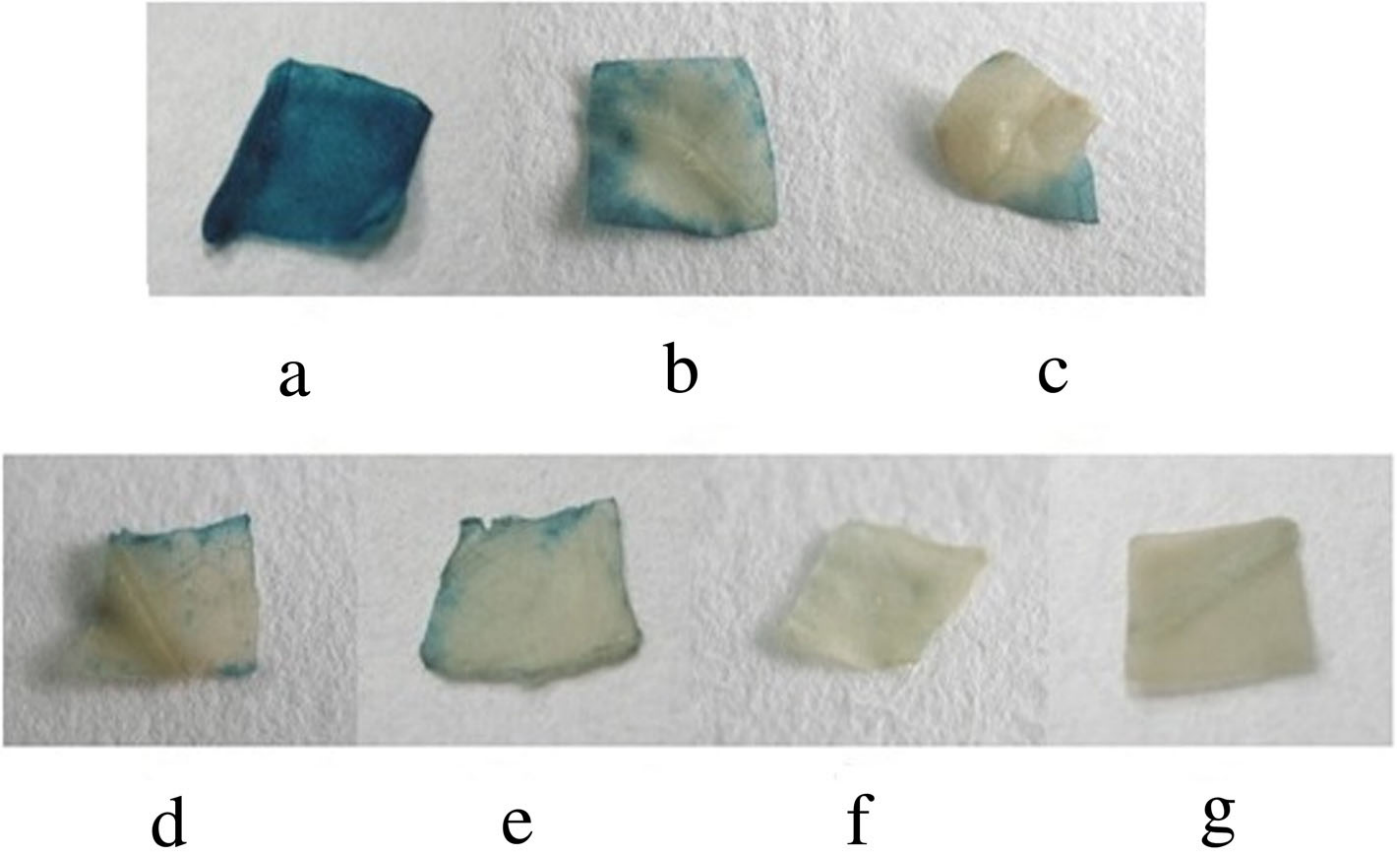
GUS expression in transgenic tobacco leaf. **a** The tobacco root transformed with plasmid pBI121; **b** The tobacco root transformed with plasmid p1200::GUS; **c** The tobacco root transformed with plasmid p900::GUS; **d** The tobacco root transformed with plasmid p600::GUS; **e** The tobacco root transformed with plasmid p400::GUS; **f** The tobacco root transformed with plasmid pBI101; **g** The wild tobacco root

**Fig. 6:**
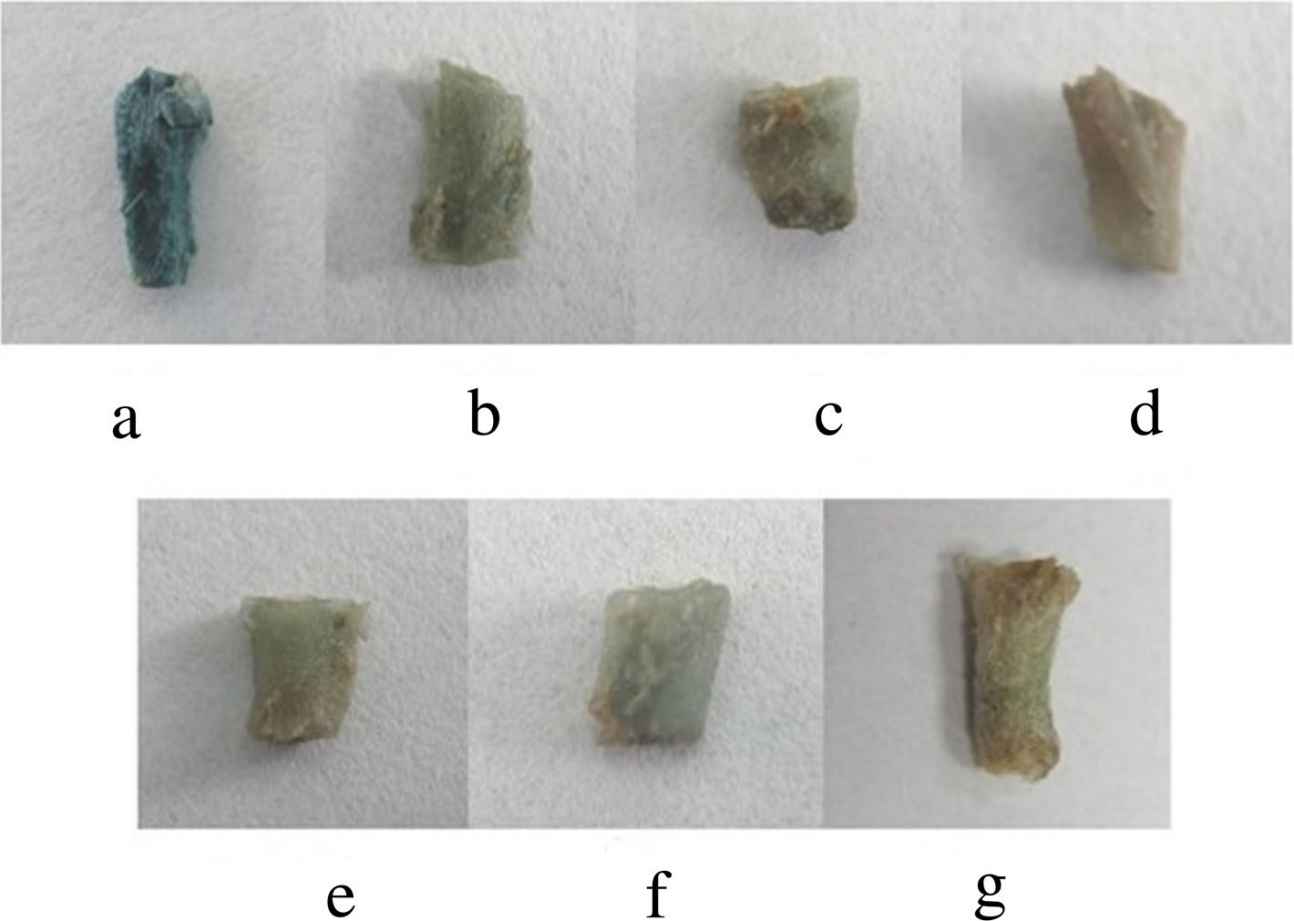
GUS expression in transgenic tobacco stem. **a** The tobacco root transformed with plasmid pBI121; **b** The tobacco root transformed with plasmid p1200::GUS; **c** The tobacco root transformed with plasmid p900::GUS; **d** The tobacco root transformed with plasmid p600::GUS; **e** The tobacco root transformed with plasmid p400::GUS; **f** The tobacco root transformed with plasmid pBI101; **g** The wild tobacco root

To investigate GUS expression driven by different lengths of *EcGS1b*-P, four different length *EcGS1b*-Ps (*EcGS1b*-P and its three 5′-deletion fragments, namely p1200, p900, p600 and p400) instead of 35 s promoter were fused with the reporter gene GUS in pBI121 vector (Fig. 3a) respectively. The fused vectors were verified by digesting the vector with *Hind*III and *Bam*HI (Fig. 3b), respectively. After, pBI121s were transformed into tobaccos, and the transgenic tobaccos were confirmed by PCR. Histochemical analysis showed that all tissues of transgenic tobaccos with pBI121 vector which had 35 s promoter (positive control) appeared in conspicuous blue (Figs. 4a, 5a, 6a), which indicated that 35 s promoter could drive GUS expression well in all tissues of transgenic tobacco. All tissues of transgenic tobaccos with pBI101 vector which had no promoter to drive GUS expression (negative control, Figs. 4f, 5f, 6f) and the wild type tobacco (Figs. 4g, 5g, 6g) were white due to lack of GUS activity, which indicated neither transgenic tobacco with no 35S promoter nor non-transgenic tobacco showed GUS activity. All roots of transgenic tobaccos with *EcGS1b*-Ps showed blue (Fig. 4b, c, d, e), which indicated they had stronger GUS activity, especially in roots transformed with longer promoters (Fig. 4b, c). When the length of the 5′-upstream region of the *EcGS1b* gene was gradually declined, GUS expression in roots also showed declination. But in leaves, all transgenic tobaccos with *EcGS1b*-Ps (Fig. 5b, c, d, e) only exhibited a little blue color at the cut edge of the blades. This indicated that GUS expressions driven by different length *EcGS1b*-Ps in leaves were all very weak. In stems, all different length promoters of *EcGS1b*-P (Fig. 6b, c, d, e) showed white, which confirmed there were no GUS expressions in these stems.

### Quantitative analysis of different length promoters of *EcGS1b*-P by 4-methylumbelliferyl-β-D-glucuronide (MUG) assays

**Fig. 7:**
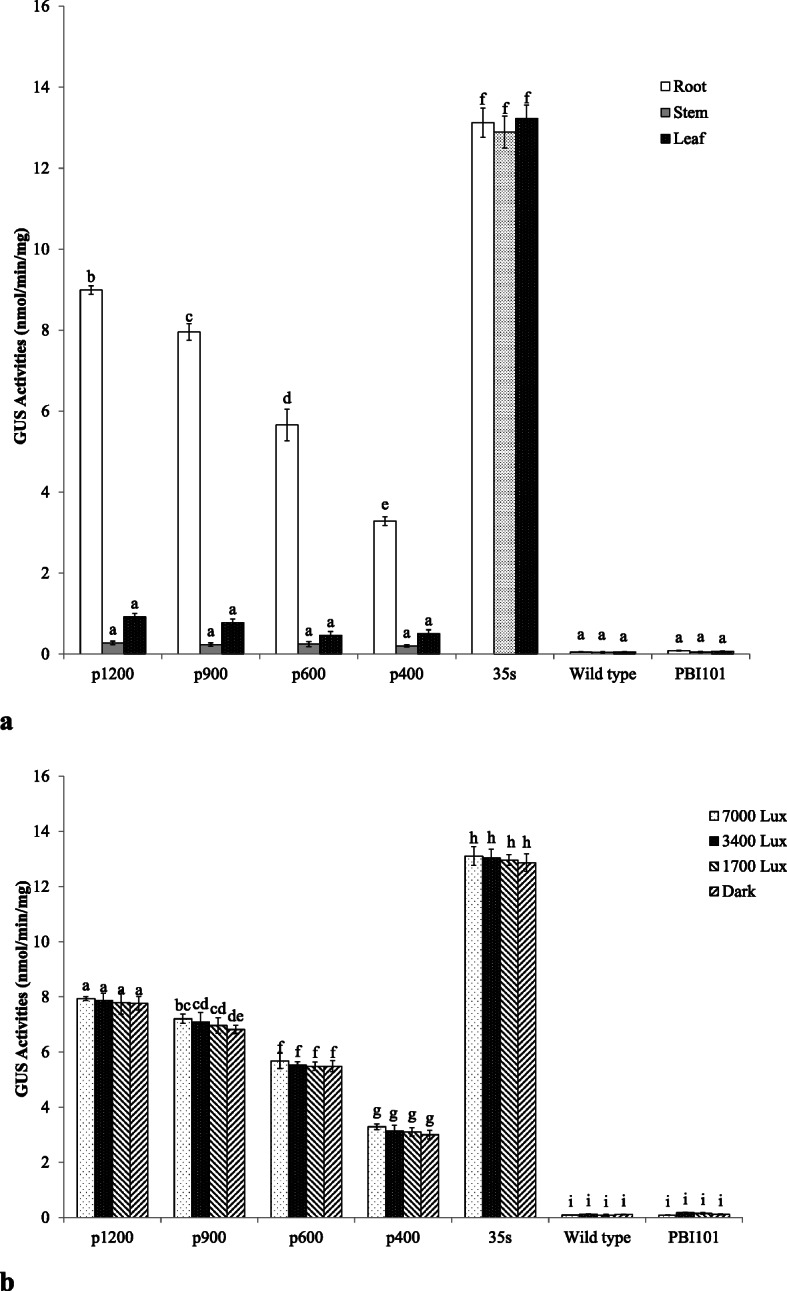
GUS activity measurement. Note: Different letters in the group indicate significant differences in data within the group (*P* < 0.01). **a** The GUS activity in one line of transgenic tobacco tissue. **b** The GUS activity in root of transgenic tobacco under different light conditions

The impact of *EcGS1b* promoter length was tested on GUS activity in transgenic tobacco by MUG assay (Fig. 7a). GUS expression in roots was the highest among three different tissues no matter which promoter of these four different length promoters was used as the GUS driver. In roots, GUS expression levels declined with decreasing of promoter length. In contrast, there was no significant difference in different length promoters both in leaves and in stems (*P* > 0.01). The GUS expression level of transgenic plant with pBI121 vector (positive control) remained the highest, even higher than that of transgenic plant with *EcGS1b*-P. There was no significant difference in different tissues in the positive control (*P* > 0.01). In the negative control, GUS activities in both wild type and the transgenic plant with pBI101 vector were almost none in roots, leaves and stems.

To determine whether GUS expression driven by *EcGS1b* gene promoter was influenced by light intensity, we examined GUS expressions in the roots of transgenic tobacco and wild type tobacco at different light intensities (7000 Lux, 3400 Lux, 1700 Lux, dark) for 3 days. As shown in Fig. 7b, no obvious variations in GUS expressions were observed at different light intensities (*P* > 0.01), and thus concluded that the GUS activity expressions were unaffected by the light intensity. In this experiment, we also found that GUS activity expressions of transgenic tobacco were related to the length of *EcGS1b*-P, confirming that the shorter the length of *EcGS1b*-P, the lower the GUS activity.

### The expression of *EcGS1b* detected by qRT-PCR

**Fig. 8:**
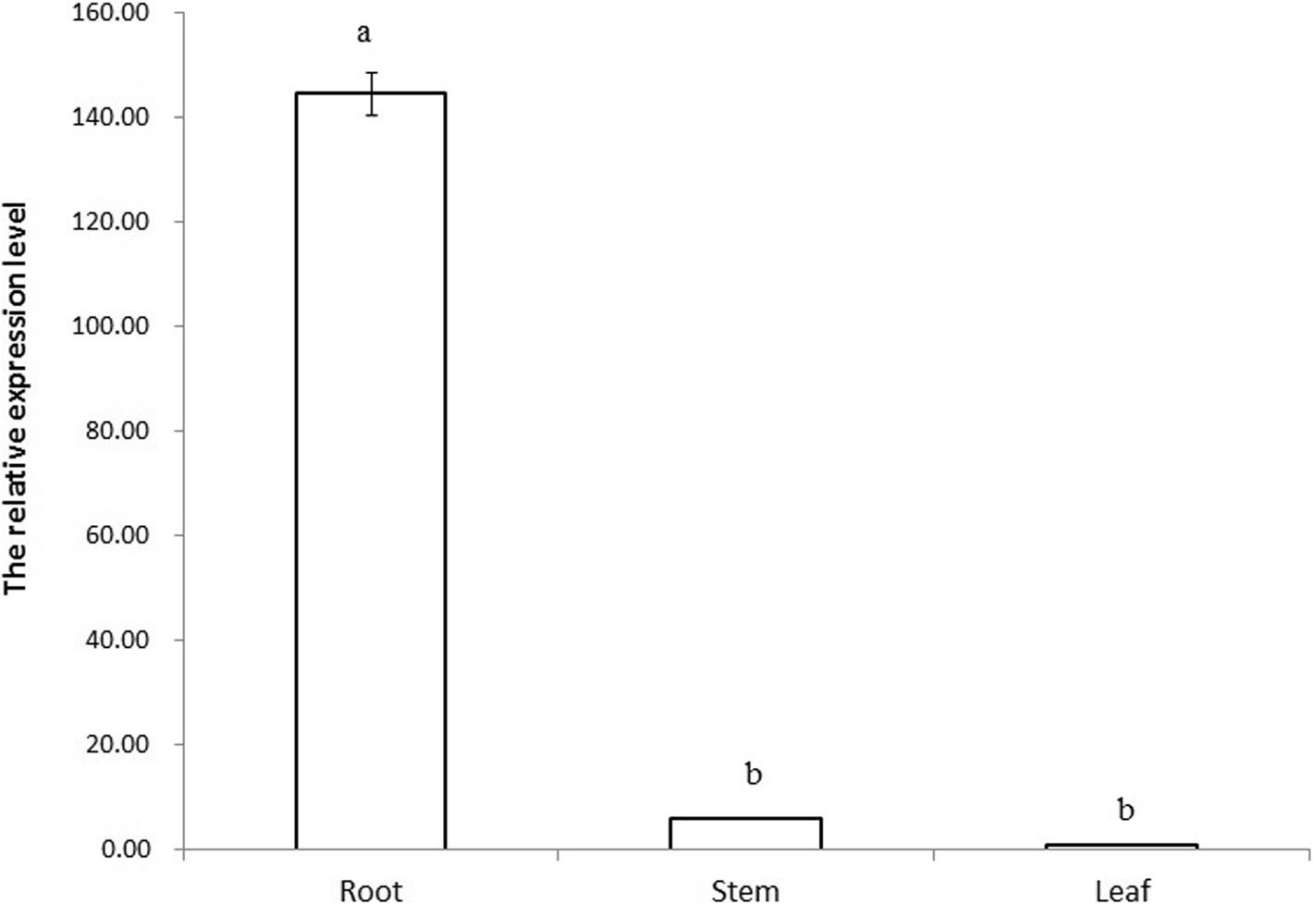
Relative expression of *EcGS1b* Gene in different tissues detected by qRT-PCR. Note: Different letters in the group indicate significant differences in data within the group (*P* < 0.01)

The total RNA of *E. crassipes* was extracted and confirmed by using an electrophoresis, and then qRT-PCR was performed to investigate the expressions of *EcGS1b* in different tissues. The results showed that the relative expression of *EcGS1b* was much higher in roots than those in stems and leaves (Fig. 8). There was no significant difference of *EcGS1b* expression in stems and leaves (*P* > 0.01).

## Discussion

Currently, the study on *E. crassipes* mainly focused on anaerobic fermentation, feed resources, fertilizer resources, water remediation and biogas production [31, 32]. There was only a little research regarding the physiological and biochemical characteristics of *E. crassipes* such as the determination of enzyme activities related to N metabolism [33–36]. Moreover, there were few papers that reported on the mechanism of *E. crassipes* at the molecular level [37, 38]. In our previous paper [38], we have cloned three GS1 genes from *E. crassipes* and studied their expressions under different N conditions. In this study, we cloned GS1b promoter from *E. crassipes* and analyzed the expression of *EcGS1b* to investigate the regulation of *EcGS1b* expression.

GS is a key enzyme in the process of N metabolism, and the efficiency of this pathway is affected by its expression [14, 39]. The effects of GS1 gene on crop growth and yield were studied by genetic engineering techniques such as by transgenic and gene silencing techniques [40, 41]. The root dry weight, grain yield, N content and resistance to adversities such as cold, drought, salinity, and biotic stresses in the transgenic wheat plants with GS gene were more than those in the wild type plants [42, 43]. The efficiency of photosynthesis could be maintained at normal level in the over-expression of GS plants under N stress conditions [44], which helps in reducing the use of N fertilizer, and making more environmental friendly and economical. The growth status of the transgenic poplar overexpressing GS1α gene of pine was better than that of the wild-type poplar [45]. But not all GS transgenic plant showed the positive effect. The effect of GS on transgenic plant was related with the coordination of N and carbon metabolism [46]. The GS activity might be downregulated via a chain of mechanisms, strongly affected by environmental conditions and metabolic imbalances [39]. Every GS has its own expression mechanism which can adapt to different N stress condition in different tissues in different plants. So studying the GS expression of *E. crassipes* and further exploring the regulation mechanism at the transcriptional level can help us to analyze GS function in order to elucidate the mechanism of efficient utilization of N in water by *E. crassipes*.

Root has many specifically expressed genes, which plays an important role in root structure and function. For instance, in Arabidopsis and Prunus, the root-specific gene *DRO1* influenced the root system architecture [47]. In Maize, 9-Lipoxygenase ZmLOX3 controlled development and maize resistance to root-knot nematodes as a root-specific suppressor [48]. A promoter can regulate the expression of its downstream gene at the transcriptional level. For example, in Arabidopsis, the promoter of *AtGln1;3* was recognized and bound to by the MYB transcription factor CCA1 to match the anticipated C supply [49, 50]. In rice, the OsMYB55 transcription factor bound to the promoter of *OsGS1;2* to initiate the transcription and mediated regulation of amino acid metabolism at high temperature [51]. Studying of root-specific promoters can facilitate analyses of gene functions in roots, further control plant development and growth [52]. The root-specific promoters also can be applied to initiate the transcriptions of root-specific genes instead of constitutive promoters such as 35 s promoter in the process of plant transgenic breeding [53]. Tissue-specific promoters enable adjustment of gene expression in a spatially controlled manner to avoid undesirable effects or excessive energetic costs to transgenic plants. So finding more tissue-specific promoters and exploring their regulation mechanisms are important for the expressions of tissue-specific genes in the breeding of transgenic plants. Much effort in mining tissue-specific promoter mainly focused on the model plants, crops and vegetables such as Arabidopsis, rice, maize, tomato and potato etc. Few studies were related with the aquatic higher plants although these aquatic higher plants are important in the remediation of eutrophication water in the ecological engineering.

In this study, *EcGS1b*-P from *E.crassipes* was isolated and analyzed with PlantCARE software. The results showed that the upstream of the promoter sequence contained many core elements such as TATA-box (− 30 bp) and CAAT-box (− 96 bp), and the distance from TATA-box or CAAT-box to TSS was consistent with that of the results described in the previous studies in other plant promoters [29]. Besides the core components TATA-box and CAAT-box, the *EcGS1b*-P also had root-specific function motif and cis-acting elements that were regulated by light, heat, hormones and temperature, which was also consistent with the previous studies [54–57]. It is worth to note that *GS1b* promoter contained root-specific expression element ROOTMOTIFTAPOX, which indicated *EcGS1b* might be a root-preferential expression gene. Previous studies have reported many root-specific promoters or root-preferential promoters that contained ROOTMOTIFTAPOX1 functional elements, which could improve the gene expression in the roots [58–61].

Next, histochemical analysis and quantification of GUS activity in transgenic tobacco were used to verify the function of *EcGS1b*-P [62–64]. We found that GUS expression level was related to the length of the promoter fragment. The longer the promoter fragment was, the higher the GUS expression level was. This indicated that there were no negative regulated elements in the *EcGS1b*-P. This was consistent with the results of Niu et al and others. Niu’s study also exhibited that blue color was declined with decreasing of *ZmPEAMT* promoter length in transgenic tobacco under no stress condition [65]. The 5′-truncated BvcPPOPs (F3, F4 and F5) also drove decreased GUS activity with the shortening of promoter length [66]. However, GUS activity was not positively correlated with the promoter length under the abiotic stress in Niu’s study [65]. Hongli Zhang also has reported that the transcription activity of 467 bp (PZ7) core fragment of *ZimPIS* promoter remained the highest in transgenic tobacco among PZ1-PZ8 [64]. In these researches, the changes in the level of GUS activity were inconsistent with those in the promoter length. This contradictory conclusion might be due to the total effect of different function elements in the promoter. Some elements might have a positive effect, while others might have a negative effect [67, 68]. So, identification and characterization of core elements and other function elements of the promoter will assist us in understanding the molecular regulation mechanism of plant gene expression and further controlling its expression by molecular technique.

Previous research studies have showed that the tissue specific promoter could increase GUS activity in this tissue of transgenic plants. The GUS activity expression of the leaf-specific *GapB* promoter in transgenic tobacco plants in leaves was higher than that in stems and roots [69]. The GUS expression of the green-tissue promoter *OrGSEp* remained high in the leaf tissues of transgenic *Arabidopsis* at different growth stages [70]. The endosperm-specific *LPAAT* promoter has specially promoted the GUS expression in the endosperm of transgenic rice plant [71]. The pollens-preferential expression promoter *OsUgp2* revealed a high GUS gene expression in the pollen of transgenic rice at the binucleate stage [72]. Our qualitative results of GUS staining results showed that the GUS activity expression driven by the *EcGS1b*-P remained the highest in the roots. There was no obvious GUS expression activity in the stems, and there was only a little in leaves transformed with longer promoters. Moreover, both quantitative results of GUS activity expression and the results of real-time RT-PCR experiments were consistent with that of the qualitative results of GUS staining. These results were also in line with the existence of ROOTMOTIFTAPOX1 elements, which was a root-specific motif in the *EcGS1b*-P that was analyzed above.

Generally, the GS gene promoter had multiple function motifs other than the root-specific motif, and so its regulatory mechanism might be complicated besides to the tissue specific, and this has been reported in previous studies. The GUS activity driven by 595*RhVI1* and 468*RhVI1* promoter was observed to be higher under light than in the dark, which might be due to the key light response elements GATA-box, I-box and GT-1box present in the promoter that responded to light in buds [73]. The GUS activity of the green tissue-specific *CsVDE* promoter was increased after exposure to high light for 4 h, but decreased after 8 h illumination, which contained many light response elements such as Box-I, GAG-motif, G-box, AE-box, GA-motif, Sp1 and GT-1 motifs [74]. Moreover, the role of downstream signaling components such as SPAI and MYC2 regulated the GUS expression of Z-box and G-box containing promoters at various wavelengths of light in different tissues [75, 76]. Although the *EcGS1b* promoter contained three light response elements BoxI, chs-CMA1a, and Sp1, the GUS activity expression level of transgenic plants with *EcGS1b* promoter was not induced by different light intensities in our study. The regulation of *EcGS1b* promoter might not be governed by light because it was a root-preferential promoter. The light response elements might be left over from genetic evolution.

## Conclusions

In this study, a 1232 bp genomic fragment upstream of *EcGS1b* sequence from *Eichhornia crassipes* has been cloned, analyzed and functionally characterized. Sequence analysis showed that there were some core elements, root specific expression elements and other functional elements. Three 5′-deletion fragments of *EcGS1b* promoter (*EcGS1b*-P) with about 400 bp, 600 bp and 900 bp length and the 1232 bp fragment were respectively used to drive the expression of GUS in tobacco. Both quantitative test and histochemical analysis revealed GUS activity was decreased with decreasing of promoter length. The GUS expressions of *EcGS1b*-P in roots were significantly higher than those in leaves and stems, indicating *EcGS1b*-P to be a root-preferential promoter. Real-time qRT-PCR expression analysis of *EcGS1b* gene also showed higher gene expression in the roots of *E.crassipes* than in stems and leaves. In all, *EcGS1b*-P is a root-preferential promoter. This study will help to elucidate the regulatory mechanisms of *EcGS1b* tissue-specific expression and further study its other regulatory mechanisms in order to utilize *E.crassipes* in remediation of eutrophic water and control its overgrowth from the point of nutrient metabolism.

## Methods

### Plant materials and growth conditions

*E.crassipes* wild type with 2–3 new leaves was obtained from the lake near the west district living area of Guangdong University of Technology. The service and management office of this university permitted our sampling. This plant material was identified by Dr. MH Fu. A voucher specimen of this material had been deposited in South China Botanical Garden. *E.crassipes* was grown hydroponically in a 4 L container with the solution containing 7 mgL^− 1^ KH_2_PO_4_, 24.5 mgL^− 1^ MgSO_4_·7H_2_O, 25.5 mgL^− 1^ KNO_3_ and 59 mgL^− 1^ Ca (NO_3_)_2_·4H_2_O, and the solution was replaced every 3 days. After it was cultivated for 1–2 weeks and new white roots grew out, the fresh new roots could be used to extract DNA.

The tobacco (*Nicotiana benthamiana*) was germinated on Murashige and Skoog (MS) medium (pH 5.8) containing 0.8% agar at 25 °C with 16 h light/8 h dark cycles and used to do plant transformation experiment. The transgenic tobacco was grown under different lighting (dark, 7000 Lux, 3400 Lux, 1700 Lux) for 3 days and then was used to perform the GUS activity experiment.

### Isolation of *EcGS1b*-P

Genomic DNA was extracted from plantlets that were grown in the hydroponic system using GV-Plant Genomic DNA Extraction Kit (GENVIEW). The quality of the genomic DNA was assessed by 1% agarose gel electrophoresis. The design of PCR specific primers (*GS1b*SP1, *GS1b*SP2, *GS1b*SP3 sequences were presented in Table 2) was based on the the *EcGS1b* sequence (NCBI GeneBank accession number KJ881169). The PCR specific primer sequences were synthesized by Sangon Biotech. The 5′-upstream promoter region of *EcGS1b* was isolated using Genome Walking Kit (Takara). The reactions were performed in 50 μL containing 5 μL DNA, 0.1 μmolL^− 1^ of each primer, 8 μL dNTP (10 mmolL^− 1^ each), 5 μL 10x PCR Buffer, 0.5 μL LA Taq (5 U/μL) with the following conditions: 94 °C for 1 min, 98 °C for 1 min, 5 cycles of 94 °C 30 s, 64.8 °C for 1 min, 72 °C for 2 min, 15 cycles of 94 °C for 30 s, 25 °C for 3 min, 72 °C for 2 min; 94 °C for 30 s, 64.8 °C for 1 min, 72 °C for 2 min; 94 °C for 30 s, 64.8 °C for 1 min, 72 °C for 2 min, enters into 94 °C for 30 s, 44 °C for 1 min, 72 °C for 2 min, and finally into 72 °C for 10 min. After that, 1 μL from the primary PCR products was used as the template for the second nested-PCR reaction and the remaining was stored at − 20 °C. The second nested PCR conditions started at 15 cycles of 94 °C for 30 s, 61.6 °C for 1 min, 72 °C for 2 min; 94 °C for 30 s, 61.6 °C for 1 min, 72 °C for 2 min; 94 °C for 30 s, 44 °C for 1 min, 72 °C for 2 min and then enters into 72 °C for 10 min. And 1 μL from the second nested-PCR reaction product was used as the third nested-PCR reaction template with the following conditions: 15 cycles of 94 °C for 30 s, 64.6 °C for 1 min, 72 °C for 2 min; 94 °C for 30 s, 64.6 °C for 1 min, 72 °C for 2 min; 94 °C for 30 s, 44 °C for 1 min, 72 °C for 2 min and then entered into 72 °C for 10 min. The product of the third nested-PCR reaction was purified using Agarose Gel DNA Purification Kit (TaKaRa) and then sequencing was performed.

**Table 2:** A list of primers used to amplify different deletions of the *EcGS1b* gene promoter

Primer	Sequence(5′-3′)
*GS1b*Sp1	GAGAGTGTCCGTGCTTTGCTTCT
*GS1b*Sp2	GCTTGAGCCATCATAGTTCCAC-3
*GS1b*Sp3	TCCCCTTGTGGTGTGTAGCAATCGC-3
F66	5′-CCCAAGCTTCATATCCCACCACCGCAT-3’
F340	5′-CCCAAGCTTCAGTGTCTGTCAGC-3’
F635	5′-CCCAAGCTTATTCATACATCTACAATC-3’
F838	5′-CCCAAGCTTCCATTGGCTGAGAATGG-3’
R1257	5′-CGGGATCCGGTTGATAAGGTCTGTG-3’
qPCR-F	5′-TTCAGGGTGACTGGAATGG-3’
qPCR-R	5′-TCCAACACGGATTGATGCT-3’
Eact-F	5′-CATTCAATGTGCCTGCCATGT-3’
Eact-R	5′-GGATAGCATGTGGAAGGGCATAG-3’

### Analysis of *EcGS1b*-P sequence

The *EcGS1b*-P was analyzed with the BLAST program of NCBI using *EcGS1b*-P sequence. Putative cis-acting regulatory elements and the transcription initiation site were predicted using TSSP-TCM software and PlantCARE (http://bioinformatics.psb.ugent.be/webtools/plantcare/html/), respectively.

### Plasmid constructions and plant transformation

A pair of primers (R1257 and F66 presented in Table 2) containing the restriction sites *Hind*III and *Bam*HI were employed to amplify the putative *EcGS1b*-P region upstream to ATG, followed by cloning the amplified fragment into the pMD19-T vector and then sequencing. After the pMD19-T vector with *EcGS1b*-P was digested by *Hind*III and *Bam*HI, the *EcGS1b*-P was sub-cloned into pBI121. Similarly, 3 more 5′-deletion promoters of *EcGS1b*-P along with 5’UTR (p900, p600 and p400) were prepared using different primers (F340, F635, and F838 in Table 2). The different promoters sub-cloned into pBI121 were named as p1200::GUS, p900::GUS, p600::GUS, and p400::GUS, respectively. These promoter constructs were integrated into *Agrobacterium tumefaciens* EHA105 and then transformed into tobacco leaf discs. The pBI121 and pBI101 vectors were also integrated into EHA105, and then transformed into tobacco respectively as the positive control and the negative control. At least two independent transgenic lines for each construct were selected for histochemical assay and GUS activity measurement.

### Histochemical assay of GUS activity

Different transgenic tobacco blades were grown in a chamber at 25 °C in dark for 2 days, and then were washed 5–6 times using sterile water. The assays of GUS expression were implemented according to the method described by Jefferson [77]. Different samples were placed in GUS staining solution at 37 °C for overnight. After dyeing, the samples were put into 75% ethanol solution for 48 h. Next, the decolorization of the leaves was observed with eyes and microscope.

### GUS activity measurement

Different tissues of transgenic tobacco were milled in liquid N and then placed in GUS extraction solution (0.05 molL^− 1^ Na_2_HPO_4_, 0.05 molL^− 1^ NaH_2_PO_4_, 0.01 molL^− 1^ EDTA, 1 mL 10% SDS, 100 μL Triton X-100, and 100 μL β-mercaptoethanol, to 100 mL H_2_O) at a ratio of 100 mg of sample to 1 mL of GUS extraction solution. The mixed samples were centrifuged at 12,000 g for 10 min, and then 50 μL supernatant was used for GUS activity. In addition, 20 μL supernatant was transferred to 1.5 mL centrifuge tube for measuring the protein concentration using the Bradford method [78]. The 250 μL reaction solution was added into GUS activity reaction containing 50 μL supernatant and 200 μL GUS reaction buffer (25 mg 4-MUG added to 25 mL GUS extraction solution) after heated at 37 °C (4-MUG buffer 2 mmolL^− 1^ 4-MUG). Then 200 μL of reaction mixture solution was added to 1.8 mL stop buffer containing 0.2 molL^− 1^ NaCO_3_ and the fluorescence level was measured immediately. The remaining reaction mixture was incubated at 37 °C for 60 min and then 200 μL of reaction mixture was added to the stop buffer. The fluorescence level of 4-methylumbelliferone (4-MU), which was the breakdown product, was determined using a fluorescence spectrophotometer at an excitation/emission wavelength of 350 nm/455 nm specifically for 4-MU. The 4-MU concentration was then determined from the standard curve. GUS activity was expressed as 4-MU nmol per minute and per milligram protein. Same tissues in at least two different lines of the same construct were mixed to do the GUS activity assay, and each was carried out three replicates. The values were expressed as means ± standard deviation. The significance of the difference was tested using the Duncan method by SPSS 11.0 software (IBM, USA).

### *EcGS1b* expression analysis by real-time qRT-PCR

Total RNA was extracted by CTAB using Plant RNA Kit (HUAYUEYANG BIOYECHNOLOGY), and then the mixed genomic DNA was removed. This was used to synthesize the first-strand cDNA in 20 μL of reaction mixture using PrimeScript™ RT reagent Kit with gDNA Eraser (Takara). Real-time PCR was performed using the SYBR Premix EX Taq™ П (Takara) with specific primers, and *E.crassipes* actin (Accession number: KC505366) was used as an internal reference gene for qRT-PCR to normalize the target gene expression (primers qPCR-F, qPCR-R, Eact-F, Eact-R given in Table 2). The real-time PCR reaction was performed on a Roche LightCycler96 PCR instrument with the following conditions: 94 °C for 5 min, 30 cycles of 94 °C for 30 s, 58 °C for 30 s, 72 °C for 1 min, and finally enters into 72 °C for 10 min. The qRT-PCR was implemented three biological replicates, and each biological replicate was performed three technical replicates. The 2^-ΔΔCT^ method was used for quantitative analysis. The values were expressed as means ± standard deviation. The significance of the difference was tested using the Duncan method by SPSS 11.0 software (IBM, USA).

## Data Availability

The *EcGS1b*-P sequence is deposited in GenBank of NCBI and assigned the accession number MT154418. This sequence can be accessible with the following link: https://www.ncbi.nlm.nih.gov/nuccore/MT154418. All other data generated or analyzed during this study are included in this published article.

## Abbreviations

GS: Glutamine synthetase
*E.crassipes*: *Eichhornia crassipes*
GUS: β-glucuronidase
qRT-PCR: Quantitative Reverse Transcription-Polymerase Chain Reaction
GOGAT: Glutamate synthase
TSS: Transcriptional start site
MUG: 4-methylumbelliferyl-β-D-glucuronide
MS: Murashige and Skoog
4-MU: 4-methylumbelliferone
